# Open questions: genomics and how far we haven’t come

**DOI:** 10.1186/1741-7007-11-109

**Published:** 2013-10-31

**Authors:** Michael Adams

**Affiliations:** 1 Department of Biochemistry, University of Georgia, Athens, GA 30602, USA

## What have we learned from doing all those genome sequences?

Go back to the genomics revolution of the last 15 years or so, and the promise of a whole new area of biology, and to me one of the most startling results of the genomic revolution is what we don’t know, rather than what we do. In general, for any genome that’s sequenced, we only really know what about a third of the genes do. For another third, we have some idea that the product might be, for example, a DNA binding protein, but we don’t know when, or where, or why it binds; or it might be a dehydrogenase, but we don’t know the substrate; and then for typically about a third of genes we have absolutely no idea of their function. And this is irrespective of the particular genome - it’s the so- called conserved hypothetical set of genes in any given genome. Obviously some genomes have been studied better than others - yeast or *Escherichia coli* genomes are clearly better charted than that of some bizarre organism from a deep sea vent. But the third to a third to a third ratio pretty much holds across all of them. So for between a third and a half of any given genome, we really have little to no idea of what the genes are doing: we may know when they’re turned on and when they’re turned off, but not what they are doing. So if we don’t know what a third or so of the genes are doing in the simplest organism, how are we ever possibly going to understand the human genome or the medical implications of its variants?

## Less about more about metabolism

You would imagine that the microbial world, which I work with, is much simpler and better-defined than the world of multicellular eukaryotes, yet there is an enormous amount we don’t understand about the simplest microbes and how they function, and the answers must lie to some extent in those parts of the genome whose functions we we still don’t know. So the fundamental open questions about the unknown parts of genomes will greatly limit other approaches to understanding how even the simplest of cells work, and a couple of examples come to mind from my own research. One is from our attempts to reconstruct global metabolism from what we know of genome sequences. This involves applying algorithms to information from genome maps to construct metabolic pathways so that we can try to predict what will happen if you grow the organism in a particular way, or perturb it in some way. It turns out they’re all quite limited, and again one reason for this must be in part the information in the genome that is not being incorporated because we don’t know what it means.

The second example is another -omic approach, metabolomics, which is aimed at identifying all the metabolites in a given cell. But even in the simplest cell you can see perhaps 2,000 metabolite peaks identified by mass spectroscopy, of which we can recognize perhaps 10%. In one sense, it is extraordinarily enlightening to realize how little we really understand biological systems, again even in the simplest cell. You have to wonder how we are ever possibly going to understand the systems biology of a human cell, whether it’s in the brain or the liver or the big toe, with this elephant in the room of genomic information that we don’t understand.

## And worse, what about non-coding RNA?

Another outstanding example of the problem of trying to assign functions to genes is the issue of non-coding RNA - RNA that does not code for proteins. This is a very active area in the microbial world now, as it is turning out that much of the genome is being transcribed whether it’s coding or not. So we have another elephant in the room, in a part of the genome that is generating noncoding RNAs of unknown function. So when you try and put all of this together to come up with true systems biology, with predictive power - since there has to be a predictive element to systems biology otherwise why do it - it’s clear we have a long long way to go in the simplest of microbes, and so we have even further to go if you’re talking about something as complex as the person that’s struggling with these unknowns.

